# miR-146a Plasma Levels Are Not Altered in Alzheimer’s Disease but Correlate With Age and Illness Severity

**DOI:** 10.3389/fnagi.2019.00366

**Published:** 2020-01-17

**Authors:** Elisabetta Maffioletti, Elena Milanesi, Abulaish Ansari, Orazio Zanetti, Samantha Galluzzi, Cristina Geroldi, Massimo Gennarelli, Luisella Bocchio-Chiavetto

**Affiliations:** ^1^Genetics Unit, IRCCS Istituto Centro San Giovanni di Dio Fatebenefratelli, Brescia, Italy; ^2^Department of Molecular and Translational Medicine, Division of Biology and Genetics, University of Brescia, Brescia, Italy; ^3^Department of Cellular and Molecular Medicine, “Victor Babes” National Institute of Pathology, Bucharest, Romania; ^4^Alzheimer’s Research Unit, Memory Clinic, IRCCS Istituto Centro San Giovanni di Dio Fatebenefratelli, Brescia, Italy; ^5^Laboratory of Alzheimer’s Neuroimaging and Epidemiology, IRCCS Istituto Centro San Giovanni di Dio Fatebenefratelli, Brescia, Italy; ^6^Faculty of Psychology, eCampus University, Como, Italy

**Keywords:** microRNA, miR-146a, Alzheimer’s disease, aging, plasma, blood

## Abstract

miR-146a is a microRNA (miRNA) involved in neuroinflammation and aging; alterations in its expression were described in Alzheimer’s disease (AD). However, most of the studies conducted so far on this miRNA included a limited number of participants and produced contradictory results. We compared miR-146a levels in plasma from 33 AD patients vs. 28 age-matched non-affected controls (CTRL) through quantitative real-time polymerase chain reaction (qRT-PCR). No difference between the case and the control group was evidenced, but a correlation was detected between miR-146a levels and subjects’ age (*p* < 0.001) as well as between miR-146a levels and patients’ Mini-Mental State Examination (MMSE) scores (*p* = 0.011), in an enlarged group of 51 AD patients and 45 CTRL supporting a role for this miRNA in aging processes and disease progression.

## Introduction

Dementia has become an important public health, social and economic issue and represents an increasing focus for policymakers, health institutions, and researchers. The most common form of dementia is late-onset Alzheimer’s disease (AD), a heterogeneous pathology caused by the combination of genetic, environmental, and lifestyle risk factors. microRNAs (miRNAs) are small non-coding RNAs which regulate gene expression by binding to the 3′-UTR of their target messenger RNAs (mRNAs) and altering their stability and/or inhibiting translation (O’Carroll and Schaefer, 2013). Almost 50% of miRNAs are expressed in the central nervous system where they play important roles in basic brain functions, in normal aging, and in different psychiatric and neurological disorders, including AD (Maffioletti et al., 2014). Studies conducted in animal models of AD and in *post-mortem* brains from patients indicated a dysregulation in the levels of several miRNAs, many of which are implicated in neuroprotective/neurodegenerative processes and target key disease genes (Reddy et al., 2017). Besides their production in cells, miRNAs are also released in several body fluids, including cerebrospinal fluid (CSF), blood and its derived products (plasma and serum). AD-related alterations in circulating miRNAs have been described by several studies, and they have been suggested as non-invasive and sensitive biomarkers which could help both in the diagnosis and prognosis of AD (Batistela et al., 2017). miR-146a (miR-146a-5p) is an immune system regulator involved in neuroinflammatory processes associated with several central nervous system diseases, including AD, and has been implicated in aging processes in animal models (Jiang et al., 2012; Deng et al., 2017). Disease-related alterations in the expression of miR-146a were described in AD, both in peripheral tissues as CSF, serum, and plasma and in *post-mortem* brains, although with contradictory results (Kiko et al., 2014; Müller et al., 2014, 2016; Denk et al., 2015; Dong et al., 2015).

Moreover, increased peripheral miR-146a levels have been shown to predict the conversion to AD in subjects with mild cognitive impairment (MCI; Ansari et al., 2019).

The aim of this study was to extend previous findings by comparing miR-146a levels in plasma samples from AD patients vs. non-affected controls, considering a much larger cohort than previous research conducted on this biological matrix (Kiko et al., 2014).

## Materials and Methods

Fifty-one AD patients were selected from a prospective study on the natural history of cognitive impairment, the Translational Outpatient Memory Clinic (TOMC) study, carried out in the outpatient facility of the National Institute for the Research and Care of AD (IRCCS Istituto Centro San Giovanni di Dio Fatebenefratelli, Brescia, Italy; Frisoni et al., 2009). The sample consisted of individuals with a diagnosis of probable AD according to the National Institute of Neurological and Communicative Disorders and Stroke (NINCDS) and the Alzheimer’s Disease and Related Disorders Association (ADRDA) criteria (McKhann et al., 1984). All patients were on stable pharmacological treatment since at least 3 months. Forty-five non-affected controls (CTRL) were also enrolled in the study. The Mini-Mental State Examination (MMSE) was performed on all study participants; for controls, a cut-off of ≥27 was applied. Age- and education-adjusted MMSE scores were calculated and used for subsequent statistical analysis. The study was approved by the local ethics committee, and all the participants and/or caregivers provided written informed consent. Blood was collected in the morning, after an overnight fast, in EDTA-containing tubes for plasma preparation. miRNAs were isolated from plasma with the kit NucleoSpin miRNA plasma (Macherey-Nagel, Düren, Germany) after having added the spike-in control cel-miR-39 (Qiagen, Hilden, Germany). The extracted miRNAs were reverse-transcribed with the TaqMan MicroRNA Reverse Transcription Kit and quantitative real-time polymerase chain reaction (qRT-PCR) was conducted on the StepOnePlus instrument using TaqMan MicroRNA Assays (Applied Biosystems, Foster City, CA, USA, assay IDs: 000468 and 000200 for miR-146a and cel-miR-39, respectively). The Ct values were normalized according to the deltaCt (dCt) method on the spike-in control cel-miR-39. The statistical analyses were performed with the software IBM SPSS Statistics; data normality was assessed through the Kolmogorov–Smirnov test. Differences in continuous and discrete variables between groups were evaluated through *t*-test (in case of significantly unequal variances, Welch’s *t*-test was applied) and chi-square test, respectively. Correlations were assessed through Pearson’s bivariate correlation analysis or, when controlling for covariates, through partial correlation.

## Results

**Figure 1:**
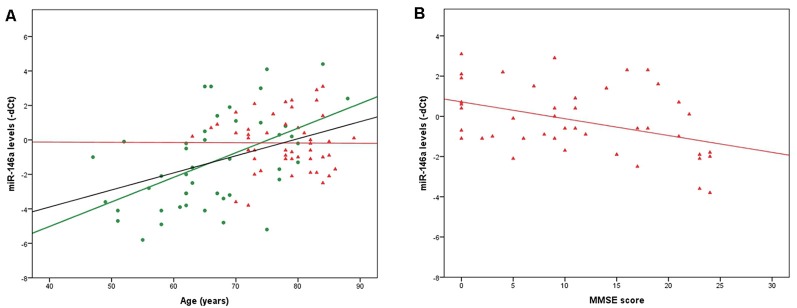
**(A)** Significant correlations between age and miR-146a levels in the whole group of CTRL + Alzheimer’s disease (AD) patients (*n* = 94, black line) and in the CTRL group (*n* = 45, green dots, green line). Although the correlation is not significant, the regression line for the AD group (*n* = 49, red triangles, red line) is also shown. **(B)** Significant correlation between Mini-Mental State Examination (MMSE) scores and miR-146a levels in the AD group (*n* = 43, red triangles, red line). For clarity, microRNA (miRNA) levels are indicated as negative deltaCts (dCts) since dCts inversely represent miRNA quantity.

qRT-PCR data (dCts) were normally distributed; we therefore conducted all the subsequent analyses applying parametric tests. Two samples were identified as outliers and excluded; the final analyses were conducted on 49 AD patients and 45 CTRL. In the whole group (AD + CTRL, *n* = 94) and in the CTRL group only (*n* = 45), a positive correlation between miR-146a levels and age was evidenced (*r* = −0.432, *p* = 1.4*10^−5^ and *r* = −0.512, *p* = 0.0003, respectively; the values of *r* are negative since the correlations were calculated on miR-146a levels expressed as dCts, which inversely represent miRNA levels). The same was not observed in the AD group only (*n* = 49, *r* = 0.002, *p* = 0.991; Figure 1A). We also highlighted sex-related differences in miR-146a levels, which were lower in females (F) compared to males (M) in the whole group (F: 0.14 ± 2.13, M: 1.60 ± 2.12; *p* = 0.002) and in the CTRL group (F: 0.31 ± 2.84, M: 2.36 ± 2.04; *p* = 0.009), but not in the AD group (*p* = 0.282). Even when controlling for sex, the correlation with age was confirmed (AD + CTRL: r = −0.403, *p* = 6.3*10^−5^; CTRL: *r* = −0.476, *p* = 0.001).

Since AD patients and CTRL were not age-matched (AD: 77.67 ± 5.91 years; CTRL: 66.33 ± 9.61 years; *p* < 2.4*10^−9^; conversely, the two groups were sex-matched: AD: 34 F/15 M; CTRL: 24 F/21 M), to test possible differences in miR-146a levels between cases and controls we selected a subgroup including 33 AD patients and 28 CTRL matched for age (AD: 74.6 ± 4.6 years, CTRL: 72.0 ± 6.7 years); the two groups were also sex-matched (AD: 25 F/8 M, CTRL: 17 F/11 M). The comparison of miR-146a levels between AD patients and CTRL revealed no significant difference (*p* = 0.884, Supplementary Figure S1). Interestingly, by taking into account the whole group, with no matching for age between cases and controls, significantly higher miR-146a levels were observed in AD patients vs. CTRL (*p* = 0.020); however, the significance was lost when age was introduced as a covariate (*p* = 0.924), indicating a confounding effect of this variable.

Finally, a negative correlation was highlighted between miR-146a levels and MMSE scores in the whole AD group (*n* = 43, since for six subjects the MMSE score was not available; *r* = 0.409, *p* = 0.007; the value of r is positive since the correlation was calculated on miR-146a levels expressed as dCts, which inversely represent miRNA levels; Figure 1B). The same was confirmed when controlling for sex (*r* = 0.422, *p* = 0.005).

## Discussion

In summary, no significant difference in plasma levels of miR-146a comparing AD patients vs. non-affected controls was detected in the present study. A positive correlation was evidenced between miR-146a levels and age, in line with animal studies which described an implication of this miRNA in aging processes, such as age-related macrophagic dysfunction, senescence and apoptosis (Jiang et al., 2012; Deng et al., 2017). An effect of sex was also observed, with females showing lower miR-146a levels. This was in line with a previous study (Zheng et al., 2018) and with other evidence indicating an influence of sex adjustment on the analysis results (Bae and Lee, 2018; Meerson et al., 2019).

A previous study reported a decrease of miR-146a levels in the plasma of AD patients compared to non-affected controls (Kiko et al., 2014). However, this investigation was conducted on a quite small sample size (10 AD patients and 10 controls) and, importantly, did not consider age as a confounding factor, although it was different between cases and controls. Since age could affect the statistical analysis when comparing groups in which this variable is different, we suggest that the influence of aging should be considered when evaluating peripheral levels of miR-146a. We also observed a negative correlation between miR-146a plasma levels and MMSE scores, indicating that, even though they do not differ between cases and controls, the levels of this miRNA could vary in relation to disease severity. It can be hypothesized that alterations in miR-146a levels during progressive illness stages could exert an influence on the expression of genes which represent important players in AD. Indeed, a validated target gene of miR-146a is the toll-like receptor (*TLR*) *2*, which encodes a primary receptor for beta-amyloid (Jurkin et al., 2010). Other AD-associated targets of miR-146a are the chemokine receptor 4 (*CXCR4*), the Fas-Associated Death Domain (*FADD*; validated targets), and the microtubule-associated protein tau (MAPT; predicted target according to TargetScan^1^). However, a limitation of the present study is represented by the fact that AD diagnosis was made clinically and lacked neuropathological or biomarker confirmation. Because of the low specificity of the NINCDS-ADRDA criteria for AD, we cannot exclude the co-occurrence of neurodegenerative conditions other than AD in our patients’ cohort.

In conclusion, the results here presented indicated no expression alterations of miR-146a in AD patients’ plasma, but revealed a correlation with age and illness severity as well as an effect of sex. Further investigations in larger cohorts are needed to replicate these findings and to clarify the role of miR-146a in aging processes and AD progression.

## Data Availability Statement

The datasets generated for this study are available on request to the corresponding author.

## Ethics Statement

The studies involving human participants were reviewed and approved by CEIOC IRCCS Istituto Centro San Giovanni di Dio Fatebenefratelli, Brescia. The patients/participants provided their written informed consent to participate in this study.

## Author Contributions

EMa and LB-C conceived the study. OZ, SG, and CG recruited and clinically characterized the patients. EMi and AA conducted the experiments. EMa, EMi and LB-C analyzed the data. EMa, LB-C and MG prepared the manuscript.

## Conflict of Interest

The authors declare that the research was conducted in the absence of any commercial or financial relationships that could be construed as a potential conflict of interest.

## Footnotes

^1^http://www.targetscan.org/vert_72/
