# Supplementary material for: miR-146a Plasma Levels Are Not Altered in Alzheimer’s Disease but Correlate With Age and Illness Severity
Source: Front Aging Neurosci. 2020 Jan 17;11:366. doi: 10.3389/fnagi.2019.00366 (PMC6978630; doi:10.3389/fnagi.2019.00366)
Supplement: Supplementary file 1 [file Data_Sheet_1.PDF]

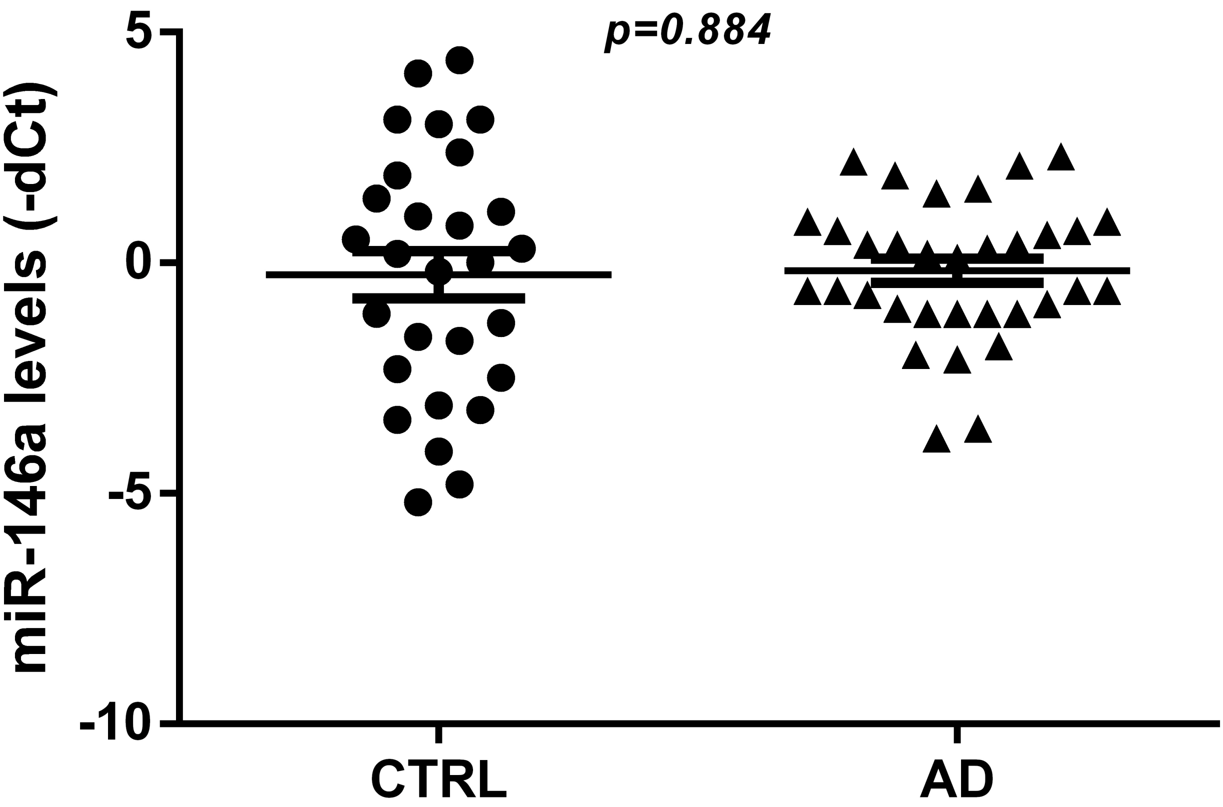

Supplementary Figure 1: miR-146a plasma levels are not different between age-matched Alzheimer's disease patients (AD, n=33) and controls (CTRL, n=28) (horizontal lines indicate mean values±standard errors). For clarity, miR-146a levels are indicated as negative dCts, since dCts inversely represent miRNA quantity.
